# Long Term Management of Rhizomania Disease—Insight Into the Changes of the *Beet necrotic yellow vein virus* RNA-3 Observed Under Resistant and Non-resistant Sugar Beet Fields

**DOI:** 10.3389/fpls.2018.00795

**Published:** 2018-07-02

**Authors:** Yann Galein, Anne Legrève, Claude Bragard

**Affiliations:** Applied Microbiology-Phytopathology, Earth & Life Institute, Université Catholique de Louvain, Louvain-la-Neuve, Belgium

**Keywords:** BNYVV, soil-borne virus, sugar beet, *Rz1* (*Holly*), *Rz2*, rhizomania, *Polymyxa betae*, nematode

## Abstract

Rhizomania disease, caused by the *Beet necrotic yellow vein virus* (BNYVV), is considered as one of the major constraints for sugar beet production, worldwide. As a result of the introgression of major resistance genes (*Holly, Rz2*) in commercially available sugar beet varieties, the virus has endured strong selection pressure since the 90s'. Understanding the virus response and diversity to sugar beet resistance is a key factor for a sustainable management of only few resistance genes. Here we report rhizomania surveys conducted in a rhizomania hot spot, the Pithiviers area (France) during a 4-year period and complementary to the study of Schirmer et al. (2005). The study aimed at evaluating the intra- and inter-field BNYVV diversity in response to different sources of resistance and over the growing season. To follow rhizomania development over the sugar beet growing season, extensive field samplings combined with field assays were performed in this study. The evolution of the BNYVV diversity was assessed at intra- and inter-field levels, with sugar beet cultivars containing different resistance genes (*Rz1, Rz1* + *Heterodera schachtii* resistance and *Rz1Rz2*). Intra-field diversity was analyzed at the beginning and the end of the growing season of each field. From more than one thousand field samples, the simultaneous presence of the different A, B and P types of BNYVV was confirmed, with 21 variants identified at positions 67–70 of the p25 tetrad. The first variant, AYHR, was found most commonly followed by SYHG. Numerous mixed infections (9.93% of the samples), mostly of B-type with P-type, have also been evidenced. Different tetrads associated with the A- or B-type were also found with a fifth RNA-genome component known to allow more aggressiveness to BNYVV on sugar beet roots. Cultivars with *Rz1*+*Rz2* resistant genes showed few root symptoms even if the BNYVV titre was quite high according to the BNYVV type present. The virus infectious potential in the soil at the end of the growing season with such cultivars was also lower despite a wider diversity at the BNYVV RNA3 sequence level. *Rz1*+*Rz2* cultivars also exhibited a lower presence of *Beet soil-borne virus* (BSBV), a *P. betae*-transmitted *Pomovirus*. Cultivars with *Rz1* and nematode (*N*) resistance genes cultivated in field infected with nematodes showed lower BNYVV titre than those with *Rz1* or *Rz1*+*Rz2* cultivars. Overall, the population structure of BNYVV in France is shown to be different from that previously evidenced in different world areas. Implications for long-term management of the resistance to rhizomania is discussed.

## Introduction

Rhizomania is one of the most challenging diseases of the sugar beet plant because of the difficulty in maintaining sustainable plant resistance sources against the causal virus, the *Beet necrotic yellow vein virus* (BNYVV), a soil-borne virus transmitted by the plasmodiophorid *Polymyxa betae* (Biancardi and Tamada, 2016). Since its discovery in (1952), the disease characterized by a constriction of the taproot with a proliferation of lateral rootlets was named accordingly “root madness” or rhizomania (rizomania) (Biancardi et al., 2002). Rhizomania is now widespread in most sugar beet-growing countries (Koenig et al., 2008; Chiba et al., 2011). Other soil-borne viruses also share the same vector and are frequently found in association with rhizomania: *Beet soil-borne virus* (BSBV) (Meunier et al., 2003), *Beet virus Q* (BVQ) (Crutzen et al., 2009), two viruses belonging to the genus *Pomovirus*, and *Beet soil-borne mosaic virus* (BSBMV) (Mahmood and Rush, 1999; Ratti et al., 2009), another *Benyvirus. Beet black scorch virus* (BBSV) (Mehrvar and Bragard, 2009) was also found associated with sugar beet exhibiting rhizomania symptoms in the USA, Iran and Inner Mongolia of China, but is considered to be transmitted in the soil to host roots by the Chytrid vector *Olpidium brassicae* (Weiland et al., 2007).

The BNYVV has a multipartite genome comprising either four or five positive sense single stranded RNAs (Tamada, 1999). The virus is able to function with the RNA1 and RNA2 molecules only for virus infection. Indeed, only these two RNAs are required for virus propagation in leaves of *Chenopodium quinoa* (Quillet et al., 1989), encoding proteins involved in viral replication, encapsidation, transmission by *P. betae* and cell-to-cell movement (Richards and Tamada, 1992; Peltier et al., 2008). The RNA3 is needed for systemic movement of the BNYVV in *Beta* species (Flobinus et al., 2016). The *p25* gene located on RNA3 was reported to enhance pathogenicity as well as general fitness and acts as an avirulence gene (*avr* gene) in resistant genotypes (Pferdmenges, 2007; Chiba et al., 2011). The *p31* gene on RNA4 is involved in transmission by the vector *P. betae*, pathogenicity and the suppression of post-trancriptional gene silencing (Guilley et al., 2009). Sometimes, the virus is also associated with an additional RNA5, which is known to enhance both symptom developments in sugar beets and aggressiveness of the virus (Tamada et al., 1996). This RNA codes the protein p26, which also contains a transcriptional activation domain (Covelli et al., 2009).

Although the variability of the BNYVV genome is considered limited in comparison with other plant viruses, a set of four consecutive amino acids, or “tetrad,” has been linked to a strong positive selection pressure on the *p25* gene (Meunier et al., 2003; Schirmer et al., 2005). This tetrad was linked with resistance-breaking (RB) events, i.e. with the emergence of symptoms in cultivars considered tolerant or resistant (Acosta-Leal and Rush, 2007; Liu and Lewellen, 2007; Koenig et al., 2008, 2009a; Acosta-Leal et al., 2010).

Since 1994, based initially on RFLP studies, three types (named A, B and P) have been described within the BNYVV species and used to differentiate isolates (Kruse et al., 1994; Koenig et al., 1997). Later on, an additional J type was proposed for Asian strains with an additional RNA5 (Schirmer et al., 2005). In 2011, based on an extensive comparison of 73 isolates of worldwide origin, Chiba et al. (2011) and Biancardi and Tamada (2016) proposed phylogenetic evolutionary pathways for BNYVV populations, with up to eight different groups with different geographical distributions.

The presence of a fifth RNA molecule, associated with the viral genome, was reported in several places in Europe, such as northern France, the UK, and Germany. The P-type was found in Pithiviers in France (Koenig et al., 1997), Kazakhstan (Koenig and Lennefors, 2000), and the UK (Ward et al., 2007) while the J-type was recorded in China and Japan (Tamada et al., 1989; Kiguchi et al., 1996). An RNA5-containing BNYVV genotype closely resembling the Chinese isolate Har4 was identified in Germany (Koenig et al., 2008). In general, no RNA5 has been associated with the A-type or the B-type (Peltier et al., 2008). In Iran, the tetrad SYHG associated with the P-type has been found without a fifth RNA in fields with severe symptoms (Mehrvar et al., 2009).

Rhizomania is primarily managed through the use of the *Rz1* gene, which confers partial resistance (McGrann et al., 2009). The *Rz1* source of resistance was initially found from *Beta vulgaris* (Holly source in California), but was broadly introgressed into sugar beet varieties (Gidner et al., 2005; Stevanato et al., 2015). It is now commonly used throughout most sugar beet-producing areas worldwide. The partial resistance of sugar beet cultivars is linked to a restriction of virus multiplication and translocation in taproots rather than in rootlets (Scholten et al., 1994; Tamada et al., 1999; Chiba et al., 2008).

The Pithiviers area, south of Paris, France was the first place where BNYVV (P-type) with the RNA5 was found and linked with a higher aggressiveness (Pferdmenges, 2007). It was also an area of intense breeding efforts with *Rz1* cultivars. In intensively cropped areas, a resistance breakdown to rhizomania began in cultivars with either *Rz1* or *Rz1*+*Rz2* due to virus evolution. It is commonly thought that such a problem is due to the emergence of resistance-breaking (RB) viral strains (Liu et al., 2005; Pferdmenges and Varrelmann, 2009). Moreover, the genetic erosion in the sugar beet due to the loss of minor genes may also play a role in the loss of resistance against the virus (Kingsnorth et al., 2002; Lennefors, 2006; Asher et al., 2009) or act synergistically to favor the emergence of such RB BNYVV isolates. The emergence of resistant-breaking isolates has now been reported in sugar beet-growing areas in Asia (Chiba et al., 2011), Europe (Bornemann and Varrelmann, 2013) and in most US production regions (Acosta-Leal and Rush, 2007), but only in Minnesota and California have these been documented to affect production at a field level.

Despite the relative diversity of BNYVV strains, mixed infections have only rarely been reported (Koenig et al., 2009a). Ratti et al. (2006) detected only a single A/B type infection out of 72 European samples. Evidence for reassortments have been reported in the UK (Ward et al., 2007), France (Koenig et al., 2009a), and localized areas in Asia (Li et al., 2008; Chiba et al., 2011).

Here we report rhizomania surveys conducted in the Pithiviers area (near Paris) during a 4-year period, complementary to the study of Schirmer et al. (2005), based on extensive field sampling and testing, combined with field assays set up to follow rhizomania development over the sugar beet growing season. The proposed study aims at evaluating the intra- and inter-field BNYVV diversity in response to different sources of resistance and over the growing season.

The diversity in 27 fields and with different sugar beet cultivars (three cultivars per field) was assessed. Different field situations around the Pithiviers area were studied at the beginning and at the end of the growing season. The different field situations are infected with nematode or not, infected with strong rhizomania and associated *Pomoviruses* or not, with high or low levels of virus and vector infectious potential, with several types of soil, with different pH of soils, with different methods of irrigation, from sugar beet production basin or not, with different previous crop grown, with different numbers of years before last beet culture. Additional samples were analyzed and information about the cultivars and the resistance profile is given. Different indicators linked to the disease were used such as the infectious potential through the Most Probable Number (MPN) of the vector and BNYVV in the soil (not shown); the presence of BNYVV, BVQ, and BSBV detected by reverse transcriptase polymerase chain reaction (RT-PCR); the BNYVV p25 amino acid tetrad, and finally, the titre of the virus in each sample by enzyme-linked immunosorbent assay (ELISA). The impact of the disease has also been measured in the field by evaluating the root symptoms severity (RS) of sugar beet root symptoms.

## Materials and methods

### Field assays and plant materials

**Figure 1:**
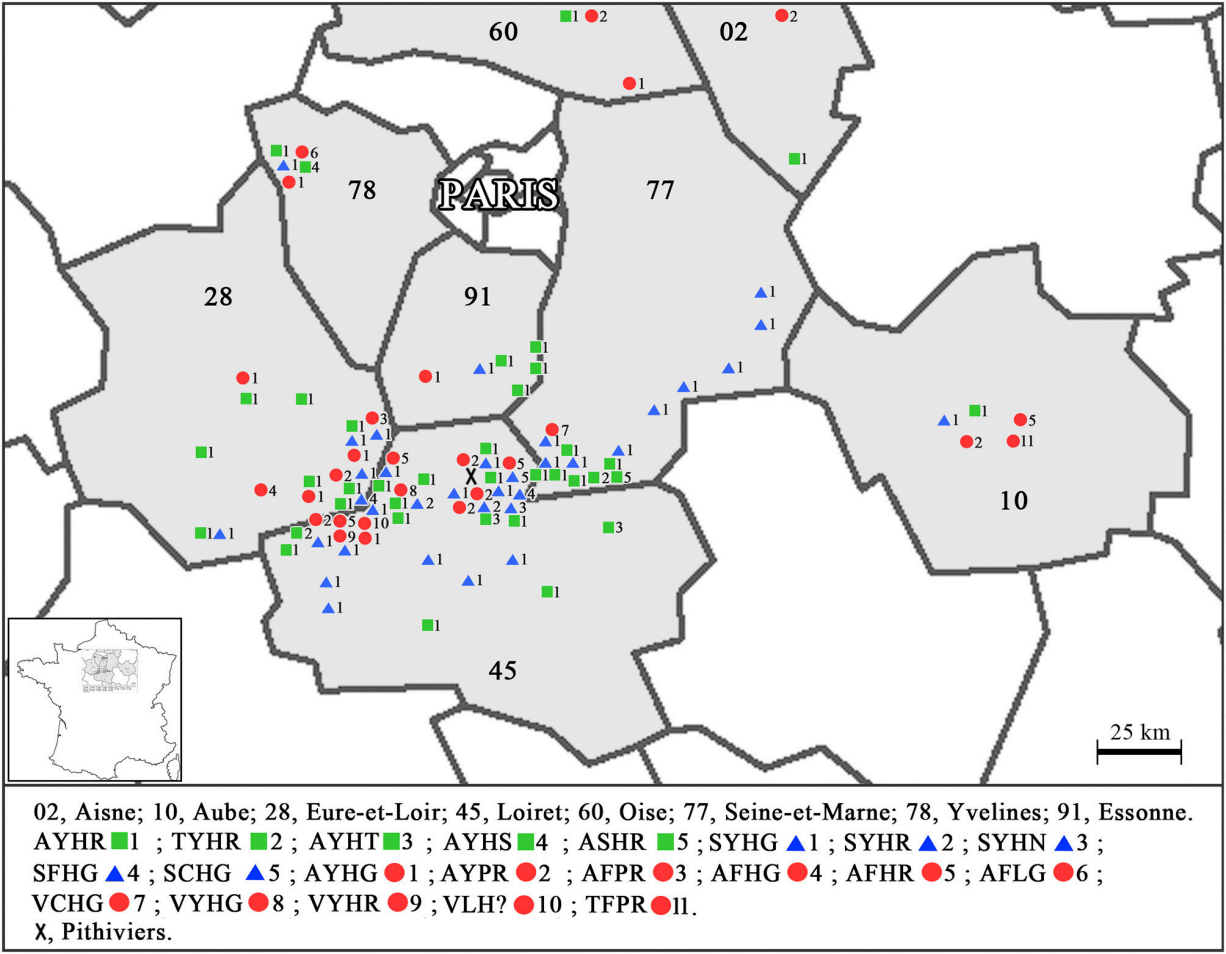
Distribution of *Beet necrotic yellow vein virus* (BNYVV) and the associated tetrads in the Pithiviers region of France. The figure shows all the tetrad diversity of BNYVV and their dispersion around Pithiviers (X). The sequences represented by a square are associated with the B-type, those represented by a circle are associated with the A-type and those represented by a triangle are associated with the BNYVV P-type.

Eight to ten field assays were conducted each year. Two French regions were studied in particular, “Loiret (with Pithiviers)” and “Seine-et-Marne,” but other regions such as “Aisne,” “Eure-et-Loire,” “Aube,” “Oise,” “Yvelines,” and “Essonne” were also investigated (Figure 1).

A total of 13 commercial cultivars (mixture of seed lots) with different resistance types were used in field assays. A frequent problem was the difficulty to get information from the breeder company regarding the homozygosity or heterozygosity of the major resistance genes in order to assess their impact on the BNYVV level or the presence of other associated viruses in plants. The cultivars were coded and are presented in Table 1. The field assays were also coded. Each field was different from the others except for the field coded F5 in Dambron 1 which is the same in year 1 and year 3. For each assay, the field was subdivided into three varietal bands and each band was subdivided into four zones. There were consequently 12 zones per field. The fields containing cyst nematodes were planted with rhizomania and nematode resistant cultivars. The minimum and maximum numbers of BCN (beet cyst nematode) eggs/larvae in 100 g of soil at initial egg density (pi) and at final egg density (pf) were the following from the set of nematode fields: the minimum pi measured was 0/0 and the maximum pi was 98/318 for the eggs/larvae, respectively; the minimum pf measured was 0/0 and the maximum pf was 85/558 for the eggs/larvae, respectively. In year 1 and year 3, there were mixed samples (one mixed sample consisted of five or six different roots) per zone pooled for the analysis. In year 2, only a single root was analyzed per zone to observe the diversity in a single root. Table 2 shows the cultivars in the diseased fields (between year 0 and year 3) that were analyzed in patches of diseased plants with symptoms. In a 150 km^2^ area corresponding to locations with severe rhizomania, targeted BNYVV genes in samples were analyzed by RT-PCR and sequencing. A total of 1058 samples were analyzed over 4 years. The root symptoms severity percentage for the root symptoms evaluation was supplied by ITB for each cultivar/plot in the field. This root symptoms severity score takes into account the percentage frequency of visible symptoms on 100 beets in this plot and the percentage of roots displaying a strong root symptom (level 2: strong symptoms, level 1: medium symptoms, level 0: no symptoms) evaluating the heterogeneity of the attack. Strong root symptom displays a heavily BNYVV infected plant displaying typical severe Rhizomania symptoms (a small “T-like” taproot with brownish vasculature and dark brown lateral roots).

**Table 1:** Locations where field assays were conducted in France and plant materials.

**Year**	**Region**	**Field code**	**Variety code**	**Variety**	**Resistance type**	**Seed company**
**FIELD ASSAYS**						
Year1	Boynes 1	F1	V1	Ludwinia	*Rz1rz1* & *Rz2rz2*	KWS
			V3	Sophia	*Rz1rz1*	KWS
			V5	Python	*Rz1rz1*	SES Vanderhave
	Boynes 2	F2	V1	Ludwinia	*Rz1rz1* & *Rz2rz2*	KWS
			V5	Python	*Rz1rz1*	SES Vanderhave
			V3	Sophia	*Rz1rz1*	KWS
	Teillay-le-Gaudin 1	F3	V8	Bering	*Rz1rz1* & *Rnematode*	Strübe
			V9	Annouschka	*Rz1rz1* & *Rnematode*	KWS
			V4	Julietta	*Rz1rz1* & *Rnematode*	KWS
	Sougy 1	F4	V4	Julietta	*Rz1rz1* & *Rnematode*	KWS
			V9	Annouschka	*Rz1rz1* & *Rnematode*	KWS
			V8	Bering	*Rz1rz1* & *Rnematode*	Strübe
	Dambron 1	F5	V4	Julietta	*Rz1rz1* & *Rnematode*	KWS
			V8	Bering	*Rz1rz1* & *Rnematode*	Strübe
			V13	Fiorenza	*Rz1rz1* & *Rnematode*	KWS
	Poupry 1	F6	V9	Annouschka	*Rz1rz1* & *Rnematode*	KWS
			V4	Julietta	*Rz1rz1* & *Rnematode*	KWS
			V8	Bering	*Rz1rz1* & *Rnematode*	Strübe
	Mondreville 1	F7	V1	Ludwinia	*Rz1rz1* & *Rz2rz2*	KWS
			V3	Sophia	*Rz1rz1*	KWS
			V5	Python	*Rz1rz1*	SES Vanderhave
	Chenou 1	F8	V5	Python	*Rz1rz1*	SES Vanderhave
			V1	Ludwinia	*Rz1rz1* & *Rz2rz2*	KWS
			V3	Sophia	*Rz1rz1*	KWS
Year2	Boynes 3	F9	V1	Ludwinia	*Rz1rz1* & *Rz2rz2*	KWS
			V5	Python	*Rz1rz1*	SES Vanderhave
			V3	Sophia	*Rz1rz1*	KWS
	Yèvres-la-Ville 1	F10	V3	Sophia	*Rz1rz1*	KWS
			V1	Ludwinia	*Rz1rz1* & *Rz2rz2*	KWS
			V5	Python	*Rz1rz1*	SES Vanderhave
	Teillay-le-Gaudin 2	F11	V4	Julietta	*Rz1rz1* & *Rnematode*	KWS
			V8	Bering	*Rz1rz1* & *Rnematode*	Strübe
			V10	Bison	*Rz1rz1* & *Rnematode*	SES Vanderhave
	Sougy 2	F12	V10	Bison	*Rz1rz1* & *Rnematode*	SES Vanderhave
			V4	Julietta	*Rz1rz1* & *Rnematode*	KWS
			V8	Bering	*Rz1rz1* & *Rnematode*	Strübe
	Dambron 2	F13	V12	Adriana	*Rz1rz1* & *Rnematode*	KWS
			V4	Julietta	*Rz1rz1* & *Rnematode*	KWS
			V8	Bering	*Rz1rz1* & *Rnematode*	Strübe
	Poupry 2	F14	V4	Julietta	*Rz1rz1* & *Rnematode*	KWS
			V8	Bering	*Rz1rz1* & *Rnematode*	Strübe
			V10	Bison	*Rz1rz1* & *Rnematode*	SES Vanderhave
	Corbeilles-en-Gatinais	F15	V3	Sophia	*Rz1rz1*	KWS
			V5	Python	*Rz1rz1*	SES Vanderhave
			V1	Ludwinia	*Rz1rz1* & *Rz2rz2*	KWS
	Mondreville 2	F16	V1	Ludwinia	*Rz1rz1* & *Rz2rz2*	KWS
			V3	Sophia	*Rz1rz1*	KWS
			V5	Python	*Rz1rz1*	SES Vanderhave
	Chenou 2	F17	V3	Sophia	*Rz1rz1*	KWS
			V1	Ludwinia	*Rz1rz1* & *Rz2rz2*	KWS
			V5	Python	*Rz1rz1*	SES Vanderhave
**FIELD ASSAYS**						
	Gironville 1	F18	V5	Python	*Rz1rz1*	SES Vanderhave
			V3	Sophia	*Rz1rz1*	KWS
			V1	Ludwinia	*Rz1rz1* & *Rz2rz2*	KWS
Year3	Boynes 4	F19	V2	Britta	*Rz1rz1* & *Rz2rz2*	KWS
			V5	Python	*Rz1rz1*	SES Vanderhave
			V3	Sophia	*Rz1rz1*	KWS
	Yèvres-le-Chatel 1	F20	V7	Rosalinda	*Rz1rz1*	KWS
			V6	Magellan	*Rz1rz1*	SES Vanderhave
			V2	Britta	*Rz1rz1* & *Rz2rz2*	KWS
	Teillay-le-Gaudin 3	F21	V11	Baobab	*Rz1rz1* & *Rnematode*	SES Vanderhave
			V4	Julietta	*Rz1rz1* & *Rnematode*	KWS
			V10	Bison	*Rz1rz1* & *Rnematode*	SES Vanderhave
	Baigneaux	F22	V11	Baobab	*Rz1rz1* & *Rnematode*	SES Vanderhave
			V4	Julietta	*Rz1rz1* & *Rnematode*	KWS
			V10	Bison	*Rz1rz1* & *Rnematode*	SES Vanderhave
	Dambron 1	F5	V4	Julietta	*Rz1rz1* & *Rnematode*	KWS
			V11	Baobab	*Rz1rz1* & *Rnematode*	SES Vanderhave
			V10	Bison	*Rz1rz1* & *Rnematode*	SES Vanderhave
	Poupry 3	F23	V11	Baobab	*Rz1rz1* & *Rnematode*	SES Vanderhave
			V4	Julietta	*Rz1rz1* & *Rnematode*	KWS
			V10	Bison	*Rz1rz1* & *Rnematode*	SES Vanderhave
	Yèvres-le-Chatel 2	F24	V3	Sophia	*Rz1rz1*	KWS
			V5	Python	*Rz1rz1*	SES Vanderhave
			V2	Britta	*Rz1rz1* & *Rz2rz2*	KWS
	Mondreville 3	F25	V5	Python	*Rz1rz1*	SES Vanderhave
			V3	Sophia	*Rz1rz1*	KWS
			V2	Britta	*Rz1rz1* & *Rz2rz2*	KWS
	Moisancelle-en-Gatinais	F26	V5	Python	*Rz1rz1*	SES Vanderhave
			V3	Sophia	*Rz1rz1*	KWS
			V2	Britta	*Rz1rz1* & *Rz2rz2*	KWS
	Gironville 2	F27	V5	Python	*Rz1rz1*	SES Vanderhave
			V2	Britta	*Rz1rz1* & *Rz2rz2*	KWS
			V3	Sophia	*Rz1rz1*	KWS

**Table 2:** Plant material from farmer's fields.

**Year**	**Region**	**Variety code**	**Variety**	**Resistance type**	**Seed company**
**FARMER'S FIELDS (PATCHES)**					
Year0	Mérouville & Sougy & Ruan & Bricy & Baigneaux & Trinay & Bucy-le-Roi & Bondaroy	V4	Julietta	*Rz1rz1* & *Rnematode*	KWS
	Chézy-sur-Marne	V5	Python	*Rz1rz1*	SES Vanderhave
	Dadonville	V17	Sporta	*Rz1rz1*	Syngenta
	Boutigny-sur-Essonnes & Saint-Pierre	V18	Ardan	*Rz1rz1*	Florimond Desprez
	Chézy-sur-Marne & Aufferville	V19	Jetta	*Rz1rz1*	Ringot Betteraves
	Mérouville & Bondaroy	V20	Zanzibar	*Rz1rz1*	SES Vanderhave
	Ramoulu	V21	Galactica	*Rz1rz1*	KWS
	Thiersanville	V22	Nordika	*Rz1rz1*	KWS
	Bondaroy	V23	Cheyenne	*Rz1rz1*	SES Vanderhave
	Bondaroy	V24	Danube	*Rz1rz1*	Florimond Desprez
	Sougy	V25	Narcos	*Rz1rz1*	Florimond Desprez
	Bondaroy	V26	Carissima	*Rz1rz1*	Betaseed
	Bondaroy	V27	Rigel	*Rz1rz1*	Betaseed
	Bondaroy	V28	Zoulou	*Rz1rz1*	Ringot Maribo
	Bondaroy	V29	Emilia	*Rz1rz1*	KWS
	Bondaroy	V30	Othello	*Rz1rz1*	Ringo Maribo
	Bondaroy	V31	Harmonia	*Rz1rz1*	Hilleshog NK
	Bucy-le-Roi & Autroche & Saint-Pierre	V32	Annalisa	*Rz1rz1* & *Rnematode*	KWS
	Bondaroy	V33	Encarta	Susceptible	Syngenta
	Bondaroy	V35	Harmonia	*Rz1rz1*	Betaseed
Year1	Yèvres-la-Ville & Bondaroy	V1	Ludwinia	*Rz1rz1* & *Rz2rz2*	KWS
	Yèvres-la-Ville & Bondaroy	V2	Britta	*Rz1rz1* & *Rz2rz2*	KWS
	Montargis & Yèvre-la-Ville & Bondaroy	V5	Python	*Rz1rz1*	SES Vanderhave
	Yèvres-la-Ville & Bondaroy	V6	Magellan	*Rz1rz1*	SES Vanderhave
	Gidy & Ruan	V8	Bering	*Rz1rz1* & *Rnematode*	Strübe
	Yèvres-la-Ville & Bondaroy	V16	Cetus	*Rz1rz1*	Deleplanque
	Yèvres-la-Ville & Bondaroy	V33	Encarta	Susceptible	Syngenta
	Yèvres-la-Ville & Bondaroy	V34	Magistral	*Rz1rz1*	SES Vanderhave
	Yèvres-la-Ville & Bondaroy	V35	Harmonia	*Rz1rz1*	Betaseed
	Yèvres-la-Ville & Bondaroy	V36	Deborah	*Rz1rz1*	KWS
	Yèvres-la-Ville & Bondaroy	V37	Antoinetta	*Rz1rz1*	KWS
Year2	Chezy	V1	Ludwinia	*Rz1rz1* & *Rz2rz2*	KWS
	Sougy	V4	Julietta	*Rz1rz1* & *Rnematode*	KWS
	Neuville-au-bois	V6	Magellan	*Rz1rz1*	SES Vanderhave
	Sougy	V10	Bison	*Rz1rz1* & *Rnematode*	SES Vanderhave
	Mayvillers	V12	Adriana	*Rz1rz1* & *Rnematode*	KWS
	Poupry	V14	Massima	*Rz1rz1* & *Rnematode*	Betaseed
	Laon	V15	Unknown	*Rz1rz1*	Unknown
	Baigneaux	V16	Cetus	*Rz1rz1*	Deleplanque
	Chezy	V28	Zoulou	*Rz1rz1*	Syngenta
	Chezy	V30	Othello	*Rz1rz1*	Ringot Maribo
	Pierrefonds	V33	Encarta	Susceptible	Syngenta
	Voué	V39	Unknown	*Rz1rz1*	Unknown
Year3	Mérouville	V2	Britta	*Rz1rz1* & *Rz2rz2*	KWS
	Teillay-le-Gaudin	V4	Julietta	*Rz1rz1* & *Rnematode*	KWS
	Sougy	V6	Magellan	*Rz1rz1*	SES Vanderhave
	Janville	V7	Rosalinda	*Rz1rz1*	KWS
	Sougy	V38	Belino	*Rz1rz1*	Florimond Desprez

### Total RNA extraction

Each rootlet was lyophilized (Lyophilisator Heto PowerDry PL6000-55 Thermo by Thermo Scientific, France) and then homogenized. The total RNA was extracted from 100 mg of homogenized root powder from each sample using the RNeasy extraction kit (Qiagen, Hilden, Germany) or similar technique.

### RT-PCR detection of the BNYVV virus

A duplex RT-PCR assay was used for the detection of RNA3 and RNA5 of BNYVV. For the RNA3, the primer pair 5′-CAGTTTATGATTTAGGGCACA-3′/5′-ATCATCATCAACACCGTCAG-3′ was used to amplify the *p25* gene by RT-PCR. For the RNA5, the primer pair 5′-ATGTTTGTTGGTCCCCCGCT-3′/5′-CGAGCCCGTAAACACCGCAT-3′ was used to amplify the *p26* gene.

### Sequencing

Before sequencing, the PCR products were purified by the MSB® Spin PCRapace Kit (Invitek GmbH, Berlin, Germany). The nucleotide sequences of the PCR products from the *p25* gene were obtained using an ABI377 Sequencer-Genetic Analyser and the “Big Dye Terminator Cycle Sequencing Kit” (Applied Biosystems). The sequences were analyzed by a Blastn search in NCBI. The Clustal W program of the Genetic Computer Group (Devereux et al., 1984) at EMBL-EBI was used to obtain multiple sequences alignment. The Expasy Proteomics Server tools (http://www.expasy.org/) were used to translate nucleotide in amino acids and the Invitrogen Vector NTI Advance 10 program (Life Technology) was used to manage the data. Each RNA3 type was characterized by SNPs (single nucleotide polymorphisms) analysis as biological markers and compared with French sequence references of each BNYVV type (A-type, AF197545; B-type, M36894; P-type, DQ682454; J-type, NC_003516).

### Quantification of BNYVV

Double antibody sandwich ELISA (DAS-ELISA) using the DSMZ kit (Leibniz Institute, Braunschweig, Germany) was carried out using polyclonal antibodies raised against BNYVV as described by Pferdmenges (2007) and following the manufacturers' instructions. The root samples coming from the field were normalized by their weight (100 mg) for the ELISA analysis. The ELISA reading was carried out at an absorbance of 405 nm. The ELISA results of the root samples were compared to a 2-fold dilution series of a positive control for the generation of a standard curve (R^2^ = 0.99). The positive control was a solution of purified BNYVV particles with a starting protein concentration of 2380 ng/ml. The ELISA values of the 2-fold dilution series of the positive control after 1 h of incubation at 37°C in the dark were 2.58, 2.09, 1.77, 1.45, 1.06, 0.80, 0.55, 0.39, 0.25, 0.17, 0.11, 0.06 for a BNYVV concentration of 2380, 1190, 595, 297.50, 148.75, 74.38, 37.19, 18.59, 9.30, 4.65, 2.32, 1.16 ng virus protein/ml buffer, respectively. The BNYVV detection limit (0 ng/ ml) resulted from a mean of tested healthy controls plus three times the standard error. Plants with estimated virus concentrations below 4 ng/ml were considered to be free of the virus as described by Paul et al. (1994).

The reason for the choice of ELISA instead of real-time RT-PCR is mostly for the robustness of the technology on samples that had to be collected from different fields, over long distances, moved and stored before processing and measuring. ELISA is offering a rather robust quantitative and comparable measure, and has been used as a standard method (e.g., Pferdmenges et al., 2009), when compared to other methodologies also used for quantification purposes (Stevanato et al., 2016). ELISA also allows a rapid quantification of the BNYVV coat protein, while real-time RT-PCR would have focused on a targeted RNA molecule from the BNYVV genome (Harju et al., 2005; Acosta-Leal et al., 2008), despite the possibility of variation between each RNA molecules (Harju et al., 2005).

## Results

### Diversity of the BNYVV p25 tetrads in the French pithiviers area

The types of p25 amino acid tetrads were evaluated over a 4-year period. The frequency of the tetrads and their location in the Pithiviers area were determined (Table 3; Figure 1, respectively). A total of 1,058 samples were evaluated and 835 were found positive for BNYVV. Amongst these BNYVV-infected samples, 482 isolates were evaluated from individual sugar beet roots (Table 3) and 353 isolates were evaluated from a composite sample made of a mix of sugar beet roots collected from a single zone in the field.

**Table 3:** Tetrad diversity observed in the Pithiviers region in France based on 482 isolates of *Beet necrotic yellow vein virus* (BNYVV) collected from year 0 to year 3.

**BNYVV infection type**	**BNYVV type according to RNA3**	**Data from (Schirmer et al., 2005)**		**This study**			
		**RNA3**	**RNA5**	**RNA3**	**RNA5**	**Freq. (%)**	**Found in varieties (this study)**
**DIVERSITY IN FRANCE**				
Single	B-type	**AYHR**_4_	+	**AYHR**_4_	+	26.45	V1, V3, **V4**, V5, V6, **V7**, **V8**, **V9**, **V10**,
							**V12**, **V14**, V16, V18, V21, V23, V25,
							V26, V28, **V32**, V35
		**AYHR**_4_	–	**AYHR**_4_	–	25.05	V1, V3, **V4**, V5, V6, **V8**, **V9**, **V10**, **V12**, V18, V19, V20, V21, V22, V30, (V33)
				TYHR	–	0.43	**V8**
				**AYHR**_4_ - TYHR	+	0.43	V1, **V4**
				**AYHR**_4_ - AYHT	–	0.21	V5
	P-type	**SYHG**_4_	+	**SYHG**_4_	+	19.01	V1, V2, V3, **V4**, V5, V6, **V9**, V16, V19,
							V20, V22, V25, V26, V28, (V33), V34,
							V36, V37
				**SYHG**_4_	–	1.51	V1, **V4**, V5
				SYHR	+	0.21	V1
				**SYHG**_4_ - SYHR	+	0.21	V35
	A-type			**AYPR**_5_	+	3.46	**V4**, V5, V6, **V7**, **V10**, V17, V24, V27,
							V29, V30, V31
				**AYPR**_5_	–	1.08	V5, **V10**
		**AFHR**_2,4_	–	**AFHR**_2,4_	+	0.43	V20
				**VCHG**_1,3_	–	0.43	V1
				**AYHG**_2,4_	–	0.21	**V4**
				AFPR	–	0.21	V20
				VYHR	+	0.21	**V4**
				**VLH?**_1_ - AFHR	+	0.21	**V4**
		ALHG	+	ND	ND	ND	Unknown
		AHHG	−	ND	ND	ND	Unknown
Mixed	B-/P-type			**AYHR**_4_ - **SYHG**_4_	+	9.93	V1, V2, V3, **V4**, V6, **V7**, **V8**, **V9**, **V10**,
							(V33), V34, V37, V38
				**AYHR**_4_ - **SYHG**_5_	–	3.89	**V4**, **V10**
				**AYHR**_4_ - **SYHG**_4_ -TYHR	+	0.21	**V4**
	B-/A-type			**AYHR**_4_ - **AYPR**_5_	+	2.16	V1, V6, **V10**, **V12**
				**AYHR**_4_ - **AYPR**_5_	–	0.65	**V4**, **V10**
				**AYHR**_4_ - **AYHG**_2,4_	+	0.21	**V8**
				**AYHR**_4_ - **AYHG**_2,5_	–	0.65	V3, V5
				**AYHR**_4_ - **AFHG**_2,4_	–	0.21	**V10**
				**AYHR**_4_ - **VCHG**_1,3_	–	0.21	V1
				**AYHR**_4_ - **AYPR**_5_ -TYHR	+	0.21	V1
				**AYHR**_4_ - **AYHG**_2,4_ - **AYPR**_5_	−	0.21	**V10**
	P-/A-type			**SYHG**_4_ - AFHR	+	0.21	V20
				**SYHG**_4_ - TFPR	–	0.21	V39
				**SYHG**_4_ - **AYPR**_5_ - AFHR	–	0.21	V39
	B-/P-/A-type			**AYHR**_4_ - **SYHG**_4_ - **AYPR**_5_	+	0.65	V6, **V10**
				**AYHR**_4_ - **SYHG**_4_ - **AYPR**_5_	–	0.43	**V10**

*Tetrads observed also by other authors: 1. Acosta-Leal and Rush, 2007; 2. Chiba et al., 2008; 3. Koenig et al., 2009b; 4. Chiba et al., 2011; 5. Bornemann and Varrelmann, 2013. Tetrads associated with resistance-breaking (RB) in Rz1 cultivars are in bold and tetrads associated with RB in wild beet B. vulgaris subsp. maritima lines are under-lined. The frequencies established are based on the 482 sequences. For the tetrad VLH?, ? means that we weren't able to identify with certitude this amino acid because there were a lot of chromatographic peaks in the position 70 when direct sequencing was made. The cultivar in parentheses corresponds to a susceptible cultivar. The under-lined cultivars correspond to Rz1Rz2 cultivars. The cultivars in bold correspond to Rz1 cultivars with nematode resistance and the others are Rz1 cultivars without the nematode resistance. ND means that we didn't found the tetrad in our study. The presence of RNA2 was also tested and was present for 63.73% of the samples. The occurrence of RNA5 with other tetrads than SYHG represents 33.77% of the samples. The total of mixed BNYVV types represents 20.25% of the samples and the total of single BNYVV types represents 79.75%*.

Amongst these 835 BNYVV positive samples, few mutations resulting in substitution of alanine with valine residue in the tetrad area of the RNA3 (0.65% of the total of 482 isolates from individual sugar beet roots) were detected. In a single sugar beet root, single infections (presence of only one BNYVV RNA3 type and only one tetrad motif) were observed in 80% of the samples. The other 20% represented mixed infection, meaning P-/B-type, B-/A-type, P-/A-type, or B-/A-/P-type infection (Table 3). In this study, 56% of the positive samples (frequency occurrence based on 835 positively infected samples) had the AYHR tetrad and a B-type RNA3 and 32% had the SYHG tetrad and a P-type RNA3 (Table 3; Figure 1). The A-type AHHG, and ALHG tetrads were not found in the root samples. Conversely, tetrads SYHG, AYHR, AYPR, AFHG, AFHR, VCHG, and VLHG linked previously with resistance-breaking events were found (Table 3). The highest tetrad diversity was found when the sugar beet cultivars with both the *Rz1* and *Rz2* resistance genes were infected with BNYVV even though the root symptoms severity was the lowest in these cultivars.

### Large co-occurrence of BNYVV A-type or B-type RNA3 with the fifth RNA (P-type)

Table 3 also shows a lot of single infections of BNYVV with a B-type (27% of the 835 positive samples) or A-type (4%) tetrads/RNA3 associated with a P-type RNA5. These particular strains with a fifth RNA account for 34% of the 482 individual sugar beet root samples with B-type AYHR being the most frequently found and then A-type AYPR in the p25. Mixed BNYVV A-/B-type infections with a fifth RNA were also observed in a single sugar beet root.

### Changes over a single growing season

**Figure 2:**
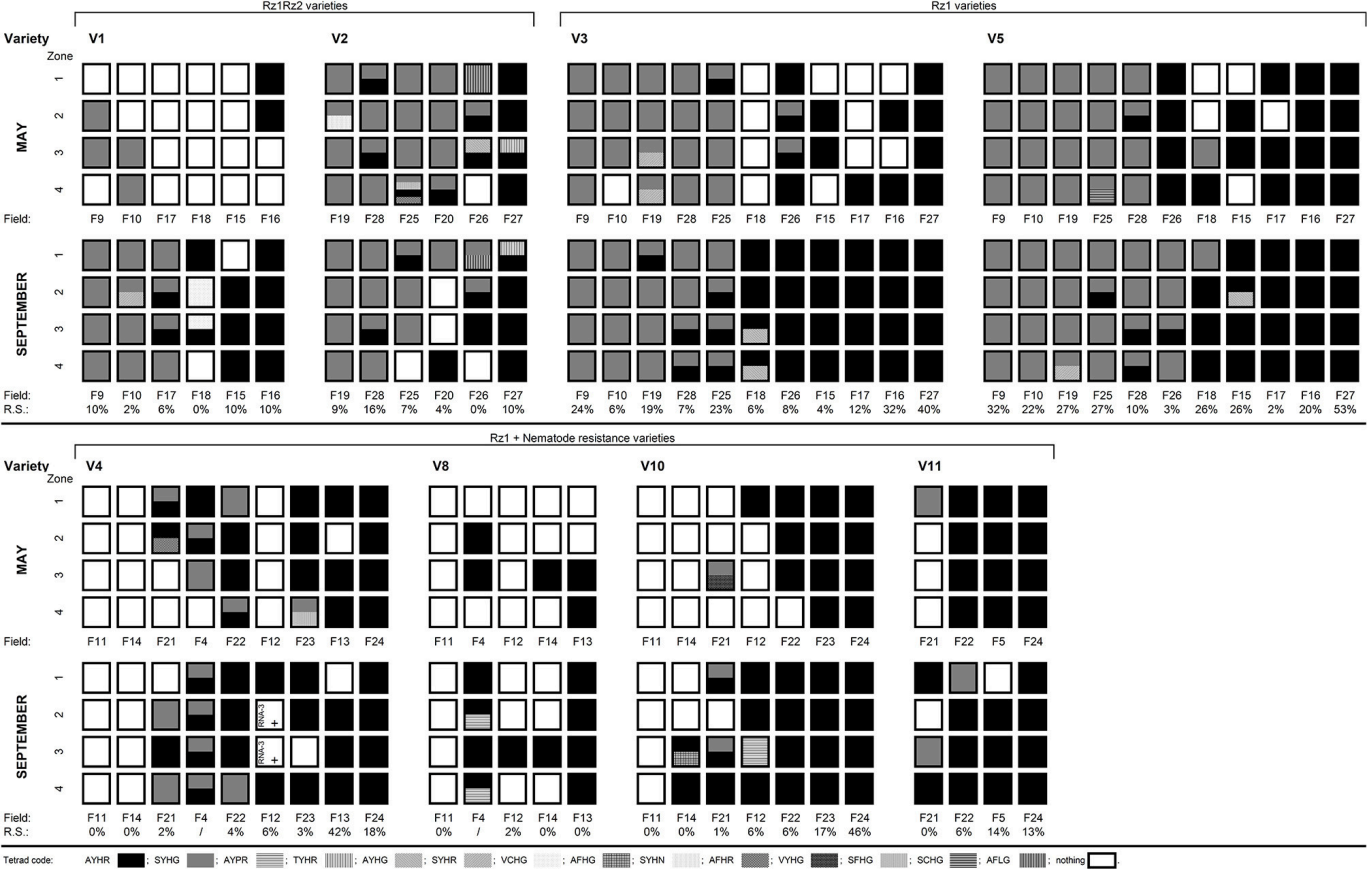
Tetrad diversity for *Beet necrotic yellow vein virus* (BNYVV) isolates by cultivar between year 1 and year 3 for samples collected from sugar beet fields in May and September in the region of Pithiviers of France. Each field was subdivided in four zones and three varietal bands. V1 and V2 were *Rz1rz1Rz2rz2* tolerant cultivars. Cultivars V3 and V5 to V7 were *Rz1rz1* tolerant cultivars. V4 and V8 to V13 were *Rz1rz1* tolerant cultivars that also included tolerance to *Heterodera schachtii*. R.S. = root symptoms severity (%) observed in September is represented by R.S based on the observation of 2 times 50 roots in each cultivar band noted with frequencies of a scale ranking the level of root symptoms (0, no symptoms; 1, average root symptom between 0 and 50%; 2, strong root symptom between 50 and 100%). /, no information.

A striking feature of the field mapping is the diversity of virus types and tetrads observed at field-level (Figure 2). Along with such diversity, changes observed between the situations early in the growing season and later on are also evident. BNYVV was detected in September in 53 zones where the virus had not been detected in May despite a systematic sampling procedure. New specific tetrads not found in May in an infected field zone were observed in September (Figure 2). Conversely, to a lesser extent, specific tetrads found in May were not observed in September. Overall, more tetrads were detectable at the end of the growing season. The number of zones per field where the virus was present also increased in September, but sometimes with different tetrads/types of virus.

### The BNYVV P-type titre is higher in plant

Based on the 20 field trials, BNYVV with a P-type RNA3 showed a higher virus content in plants (mean ELISA value of 1.75 for *Rz1rz1Rz2rz2* and 1.81 for *Rz1rz1*) than BNYVV with B-type RNA3 (mean ELISA value of 0.42, 0.49 and 0.51 for *Rz1rz1Rz2rz2, Rz1rz1*, and *Rz1rz1*+*N*, respectively) or mixed-type RNA3 (mean ELISA value of 0.63, 1.04 and 0.46 for *Rz1rz1Rz2rz2, Rz1rz1*, and *Rz1rz1*+*N*, respectively) in the cultivars (Table 4).

**Table 4:** Enzyme linked immunosorbent assay (ELISA) values for *Beet necrotic yellow vein virus* resistance cultivars grown in France.

**Resistance background**	**BNYVV RNA3 type**	**Mean ELISA ± *SD***	***n***	Fisher's LSD^**^
				
Rz1rz1Rz2rz2	P	1.75 ± 0.15	3	c
	B	0.42 ± 0.29	2	ab
	Type mixture	0.63 ± 0.57	8	ab
^*^Rz1rz1 without N	P	1.81 ± 0.74	8	c
	B	0.49 ± 0.24	8	a
	Type mixture	1.04 ± 0.63	10	b
^*^Rz1rz1 with N	P	Not observed	0	
	B	0.51 ± 0.23	13	a
	Type mixture	0.46 ± 0.14	7	a

*The average ELISA values are represented as the average optical density of the four zones (three replicates per zone minus twice the blank) more or less a standard deviation (SD). The average of the average ELISA values is then calculated for each type of resistance. There is a statistically significant difference between the comparisons among the three different categories of means at P = 0.05 when F obs> F-test (Fisher's LSD test, homogeneous group, alpha = 0.05)*.

* *Rz1rz1 cultivars without N (nematode resistance); Rz1rz1 cultivars with N (nematode resistance)*.

** *Means followed by same letter are not significantly different according to Fischer's LSD test (P = 0.05)*.

Statistically, the highest ELISA value was measured in *Rz1rz1Rz2rz2* and *Rz1rz1* cultivars when infected with the P-type and then in *Rz1rz1* cultivars when infected with the mixed-type. The intermediate ELISA value was measured in *Rz1rz1Rz2rz2* cultivars when infected with the B-type or the mixed-type. The lowest ELISA value was measured in *Rz1rz1* cultivars when infected with the B-type and in the *Rz1rz1*+*N* cultivars when infected with the mixed-type or the B-type (Table 4).

Moreover, at the end of the growing season, there were statistically no significant differences (Fisher's LSD, homogenous group, alpha = 0.05) between the ELISA value of the B-type tetrads with the RNA5 or without RNA5 (data not shown). However, the frequency of the B-type RNA3 in the field (52%) was higher than the frequency of the P-type RNA3 (21%), the frequency of the A-type RNA3 (6%) and the one of the mixed-type RNA3 (20%) (Table 3; Figure 2).

### Mixed BNYVV types

Mixed infections with various BNYVV types were observed in the collected sugar beet taproot. In the field between year 1 and year 3, the tetrads AYHR and SYHG were the ones that were most frequently found in mixed infections. All three RNA3 types (A-type, B-type, P-type) could also be found in some individual samples (Table 3).

When there was mixed-RNA3 B- & P-type infection, the recorded root symptoms severity (RS) was the lowest in *Rz1rz1Rz2rz2* (mean RS: 5.62%) and *Rz1rz1*+*N* (rhizomania and nematode resistance) (mean RS: 2.71%) cultivars. It was also the lowest compared to single type infection in all kinds of cultivars (Table 5). The RS of the *Rz1rz1Rz2rz2* (mean RS: 9.5%) cultivars infected with the P-type alone, the RS of the *Rz1rz1Rz2rz2* (mean RS: 10%) cultivars and the RS of the *Rz1rz1*+*N* (mean RS: 12.85%) cultivars infected with the B-type alone are statistically similar. This group has an intermediate RS that is significantly different from the previous group and the next one. The highest RS was measured in *Rz1rz1* cultivars whatever the kind of infection (P-type alone, B-type alone or mixed-type infection). The range of the mean RS is for this group between 17.4% for the mixed-type infection and 21.37% for the single type infection.

**Table 5:** Root symptoms severity for *Beet necrotic yellow vein virus* resistance cultivars grown in France.

**Resistance background**	**BNYVV RNA3 type**	**Mean root symptoms severity ± *SD* (%)**	***n***	Fisher's LSD^**^
Rz1rz1Rz2rz2	/	7 ± 4.80	12	a
^*^Rz1rz1 without N	/	19.12 ± 12.82	24	b
^*^Rz1rz1 with N	/	9.30 ± 13.19	20	a
Rz1rz1Rz2rz2	P	9.5 ± 0.71	2	ab
^*^Rz1rz1 without N	P	19 ± 9.34	6	a
^*^Rz1rz1 with N	P	Not observed	0	/
Rz1rz1Rz2rz2	B	10 ± 0	2	ab
^*^Rz1rz1 without N	B	21.37 ± 18.54	8	a
^*^Rz1rz1 with N	B	12.85 ± 15.27	13	ab
Rz1rz1Rz2rz2	Type mixture	5.62 ± 5.45	8	b
^*^Rz1rz1 without N	Type mixture	17.4 ± 9.81	10	a
^*^Rz1rz1 with N	Type mixture	2.71 ± 2.63	7	b

*There is a statistically significant difference between the comparisons among the three different categories of means at P = 0.05 when F obs> F test (Fisher's LSD test, homogeneous group, alpha = 0.05)*.

* *Rz1rz1 cultivars without N (nematode resistance); Rz1rz1 cultivars with N (nematode resistance)*.

** *Means followed by same letter are not significantly different according to Fischer's LSD test (P = 0.05)*.

The P-type alone was not detected from individual roots in fields infested with nematodes, but was detected in mixed infections (Tables 4, 5; Figure 2). Generally, the P-type alone was less present in the fields with nematodes.

Overall, cultivars infected with the AYHR tetrad without RNA5 showed the lowest mean RS (11%) followed by cultivars infected either by mixed AYHR & SYHG tetrads with RNA5 (12%) or by the SYHG tetrad alone with RNA5 (15%). The highest mean RS (16%) was measured in cultivars infected by strains showing the tetrad AYHR with RNA5 (Table 6). There were statistically significant differences between these three groups, independently of the cultivar.

**Table 6:** Mean root symptoms severity values for BNYVV tetrads with or without RNA5 independently of the cultivar.

**BNYVV RNA3 tetrad**	**RNA5**	**Mean root symptoms severity ± *SD* (%)**	***n***	Fisher's LSD^*^
AYHR	No	11.27 ± 12.62	51	a
AYHR-SYHG mixed	Yes	11.58 ± 9.35	24	ab
SYHG	Yes	15.03 ± 9.25	58	ab
AYHR	Yes	16.33 ± 15.87	61	b

*There is statistically a significant difference between the comparisons among the different categories of means at P = 0.05 when F obs> F-test (Fisher's LSD test, homogeneous group, alpha = 0.05)*.

* *Means followed by same letter are not significantly different according to Fischer's LSD test (P = 0.05)*.

## Discussion

The BNYVV diversity in the Pithiviers region, south of Paris in France, was found to be very different from other resistance-breaking associated epidemic foci. The Pithiviers region has been repeatedly described as an intense sugar beet breeding area (De Biaggi et al., 2003). In line with Schirmer et al. (2005) who previously pointed out the presence of various BNYVV types and tetrads, this study emphasizes the increasing diversity of the virus both at the area and field levels, thereby underlining the complexity of the disease. During a single growing season, independently of the cultivar background, different p25 tetrads appeared and others disappeared. There was as such a tetrad selection through the cultivar background. Before 2005, three types and five tetrads (four hypervariable amino acids in the ARN3-coded p25) known in the area, based on 40 isolates, were detected: the B-type AYHR tetrad (54%), followed by P-type SYHG tetrad (32%) and A-type AFHR (3%), AHHG (3%), and ALHG tetrad (6%) (Schirmer et al., 2005). In contrast, a much higher diversity (21 tetrads in total from 3 BNYVV types) was assessed in this study (Table 3; Figure 1).

### The situation in pithiviers is different

The presence of A-, B-, and P-type BNYVV in the Pithiviers area is quite unique. This situation is distinct from that of other sugar beet production regions: the strong selection pressure created by the widespread use of resistant cultivars, associated with an intense breeding area (De Biaggi et al., 2003), seems to favor the occurrence of parallel mutations in individual virus populations, calling into question how long *Rz1* resistance will be useful (Acosta-Leal et al., 2010; Bornemann and Varrelmann, 2013). Chiba et al. (2011) have proposed an evolutionary pathway reconstructing the spread and the occurrence of resistance breaking isolates of BNYVV in different sugar beet-growing areas (VCHG, VLHG, ALHG, AHHG, ACHG, AFHG, SYHG, AQHG, AYRV tetrads, a hypervariable region in the p25). The resistant-breaking (RB) selection can be found in association with a single mutation in the tetrad (Bornemann and Varrelmann, 2013) and also with other mutations along the *p25* (Klein et al., 2007). Usually, both the former wild type (WT) and new resistant-breaking (RB) tetrads are geographically localized. In the USA, an RB zone without a fifth RNA is linked with a single mutation in *p25* (Acosta-Leal et al., 2010).

Rhizomania in France around Pithiviers is different from other regions with resistant-breaking strains in the USA, Europe (Acosta-Leal and Rush, 2007; McGrann et al., 2009), and Asia (Chiba et al., 2011), in that much of the known world virus diversity can be found in only a 150 km^2^ production area. Nine recognized and putative (SYHG, AYHG, AFHG, AFHR, AYPR, VLHG, VCHG, TYHR, and AYHR) *Rz1*- and/or *Rz2*-RB BNYVV isolates have been documented in the area. Furthermore, the simultaneous presence of at least three BNYVV types along with evidence for reassortments (Koenig et al., 2008) between isolates highlights the need to manage available sources of resistance as efficiently as possible. Peltier and colleagues also found RNA5 associated with European BNYVV A-, B-, and SYHG P-types (Peltier et al., 2008). Despite reduced symptoms, our data showed that *Rz1*+*Rz2* cultivars did not offer a total protection each time, with both an increase in the number of plots infected by the virus between the beginning and the end of the sugar beet growing season, and sometimes the appearance of new tetrad variants. Consequently, there is a risk for the emergence of new *Rz1*+*Rz2* breaking strains. So far no other resistance genes than *Rz1* and *Rz2* have been discovered, and their use should be managed. Before reaching such conclusion, it would have been useful to ascertain the allelic status of *Rz1, Rz2* and nematode resistance sources of the different sugar beet varieties used in this study.

### Spatial diversity at regional and field level

Besides these observations, the high frequencies (years 1–3) of BNYVV P-type (SYHG tetrad) or BNYVV B-type (AYHR tetrad) were striking when compared to the very low frequencies measured for known BNYVV A-type RB isolates with the tetrads VCHG or others. Based on our field-based trials, it was possible to determine that the diversity was found not only at the regional level but also at the field-scale level, and both at the beginning and at the end of the growing season. Using deep sequencing and bioassays, Bornemann and Varrelmann found that sugar beet genotypes induce a strong selective effect on the accumulation of different p25 tetrad variants. RB tetrad mutations are selected with a loss of relative fitness, with the exception of P-type (Bornemann and Varrelmann, 2013). Some genotype would select for the development of viral strains and other genotype would support the development of other unrelated specific viral strains. The same phenomenon was observed in our study but at the field level. The diversity observed in the Pithiviers area could be a reflection of the major breeding and rhizomania resistance research effort that has taken place in the area since the 1970s (De Biaggi et al., 2003).

In general, a tetrad diversity was observed intra-field (within the same field) and a cultivar resistance difference between the three cultivars in the same field was also observed regarding these tetrads. Schirmer et al. found only little tetrad diversity in France. The A-, B-, and P-type were also found in their study, but only the tetrad SYHG, AYHR, AFHR, AHHG, and ALHG was described (Table 3) (Schirmer et al., 2005). Variability is a key factor for RNA virus pathogenicity, where adaptation to changing situations serves to preserve genetic robustness and maintain fitness despite the presence of mutations in the genome (Schirmer et al., 2005). According to Bornemann and Varrelmann, different sugar beet genotypes would support the development of divergent viral populations (Bornemann and Varrelmann, 2013). In the present study, we found higher tetrad diversity in each type (Figure 1). The tetrad AYPR was also associated with clear resistance-breaking events. Several tetrads usually associated with the A-type or the B-type were also found with the fifth RNA.

### Higher accumulation of BNYVV P-type

In the present study, the P-type associated RNA5 was detected in 52% of the 835 BNYVV positive samples. The relationship between the tetrad SYHG, the virus aggressiveness and the sugar beet susceptibility is still not fully clear. By ELISA, BNYVV P-type accumulates at much higher levels in resistant, tolerant and susceptible sugar beet cultivars than BNYVV B-type. This was also observed by Heijbroek et al. where the percentage of plants in which the virus reached only a low concentration was much lower in P-type than in A- or B-type infections (Heijbroek et al., 1999). Therefore, one could speculate that the P-type move faster in the plant than the B-type. Surprisingly, such quantification and root symptoms severity measures indicate a competition between both types, based on the measures on both single and mixed infection within an individual beet. The BNYVV mixed infection level in a single sugar beet was lower than in P-type single infection level or B-type single infection level. It seems that there is a modulation of the different BNYVV RNAs in the plant according to the type present. The tetrad SYHG was also linked to a very high level of ELISA value and consequently CP content. However, the tetrad AYHR was linked to a lower content of CP in the plant but higher in case of mixed infections. The competition between B- & P-type is less evident when comparing the ELISA data rather than the RS.

### RNA5 preferentially associated with RNA3 B-type or A-type

Ratti et al. (2009) showed that BSBMV RNA3 can be replicated and encapsidated when co-inoculated with BNYVV RNA1 and 2 in *Beta macrocarpa*. Long-distance movement was observed indicating that BSBMV RNA-3 could substitute BNYVV RNA-3 for systemic spread, even though the p29 encoded by BSBMV RNA-3 is much closer to the BNYVV RNA-5-encoded p26 than to BNYVV RNA-3-encoded p25. In this study, mixed infections of tetrads were related to the A-type and/or B-type and/or P-type of BNYVV RNA3 in individual sugar beet taproot. Approximately 20% had a fifth RNA with a tetrad associated to the A-type and/or B-type RNA3 while the P-type RNA3 was associated with the P-type RNA5 for 100% most of the time. This indicates a stronger presence of A-type or B-type associated with P-type RNA5 than previously indicated (Koenig et al., 2009a). Based on Ratti et al. (2009)'s results one could then speculate that the BNYVV mixed type infection could also occur in a single plant. Meunier et al. (2005) suggested earlier that a recombination event had taken place within the RNA2 between the BNYVV B-type and A-type. In 2009, Koenig et al. also suggested a BNYVV genome reassortment in several resistant sugar beet cultivars with strong rhizomania symptoms (Koenig et al., 2009a). The RNA5 was usually detected either with A-type RNAs 1–4 or with a mixture of B-type and P-type RNAs. However the distinction between mixed BNYVV type infection and reassortment is not easy to formally demonstrate. In Spain, particular tetrads (VCHG) linked to the BNYVV A-type were responsible for resistance-breaking and the fifth RNA was not found (Koenig et al., 2005; Schirmer et al., 2005). In Italy, only the A-type without the fifth RNA was found (Koenig and Lennefors, 2000).

### *Rz1*+*N* cultivars grown in field infected with nematodes showed lower virus content

In 1995, an experimental hybrid (obtained by different pollinators crossed with the same monogerm CMS) with R22 (Lewellen and Wrona, 1997; Lewellen, 2000) was grown in an Imperial Valley test under rhizomania conditions in comparison to “Rhizosen” (*Rz1* Holly Hybrids cultivar) and a rhizomania susceptible commercial cultivar “HH41.” R22 was developed from a cross between a single sugar beet line (C37) and 60 sea beet accessions (Lewellen, 1992). R22 and R22 hybrids seemed to express higher resistance to rhizomania than that conditioned solely by *Rz1* (Biancardi et al., 2012). It was unclear whether this higher resistance was due to improved resistance to rhizomania or to some other pest or disease (Biancardi et al., 2012). When *Rz1*+*N* cultivars were grown in field infected with nematodes, the ELISA values were lower than within *Rz1* or *Rz1Rz2* cultivars (Table 3). This was observed in fields infected either with the BNYVV B-type or with a mixed-type. Surprisingly, the different fields infected with nematodes showed almost no P-type in the different cultivars, especially the more rhizomania resistant ones (Figure 2). Moreover, the root symptoms severity in *Rz1*+*N* cultivars was at the same level as *Rz1Rz2* cultivars independently of the BNYVV type observed (Table 5). Those results suggest that the concomitance of the nematodes in the soil and the presence of nematode resistance in the *Rz1* cultivar allow a drastic reduction of the rhizomania titre in the plant during the growing season. One could speculate either that the interaction between the nematode and the nematode resistance gene stimulate the SAR in the plant or other resistant mechanism enhancing performance and/or disease resistance against the BNYVV or that the nematode in the soil is in competition with the viruliferous *P. betae*. Incidentally, this last hypothesis was observed in the virus MPN results (data not shown). Over the sugar beet growing season, the viruliferous *P. betae* multiplied less under the *Rz1*+*N* cultivars than under the *Rz1* or *Rz1Rz2* cultivars. Moreover, the *P. betae* themselves or other pathogens (bacteria, nematode) could be involved in the stimulation of the plant defense mechanism, reducing infection of sugar beets by *P. betae* as Desoignies et al. (2013) showed with lipopeptides of *Bacillus amylolequifaciens*.

## Accession number

The partial BNYVV RNA3 sequences in this study have been deposited in GenBank. GenBank accession numbers for partial RNA sequences are MG839229 to MG839249 for the BNYVV RNA3-coded p25 of isolates AYHR, TYHR, ASHR, AYHT, AYHS, SYHG, SYHR, SYHN, SFHG, SCHG, AYPR, AFPR, TFPR, AYHG, AFHG, AFHR, AFLG, VCHG, VYHG, VYHR, VLHX, respectively.

## Author contributions

All authors listed have made substantial, direct, and intellectual contribution to the work, and approved it for publication. YG and CB conceived the study. YG conducted the assays. All authors analyzed the data, and wrote the manuscript. All authors have read and approved the final manuscript.

### Conflict of interest statement

The authors declare that the research was conducted in the absence of any commercial or financial relationships that could be construed as a potential conflict of interest.
